# Metal-Triggered FAD Reduction in d-2-Hydroxyglutarate Dehydrogenase from *Pseudomonas aeruginosa* PAO1

**DOI:** 10.1021/acsbiomedchemau.4c00108

**Published:** 2024-12-06

**Authors:** Joanna Afokai Quaye, Giovanni Gadda

**Affiliations:** † Departments of Chemistry, Georgia State University, Atlanta, Georgia 30302-3965, United States of America; ‡ Biology, Georgia State University, Atlanta, Georgia 30302-3965, United States of America; § The Center for Diagnostics and Therapeutics, Georgia State University, Atlanta, Georgia 30302-3965, United States of America

**Keywords:** *Pseudomonas aeruginosa*d-2-hydroxyglutarate dehydrogenase, solvent isotope effects, viscosity effect, Zn^2+^, metallo flavoprotein, flavin

## Abstract

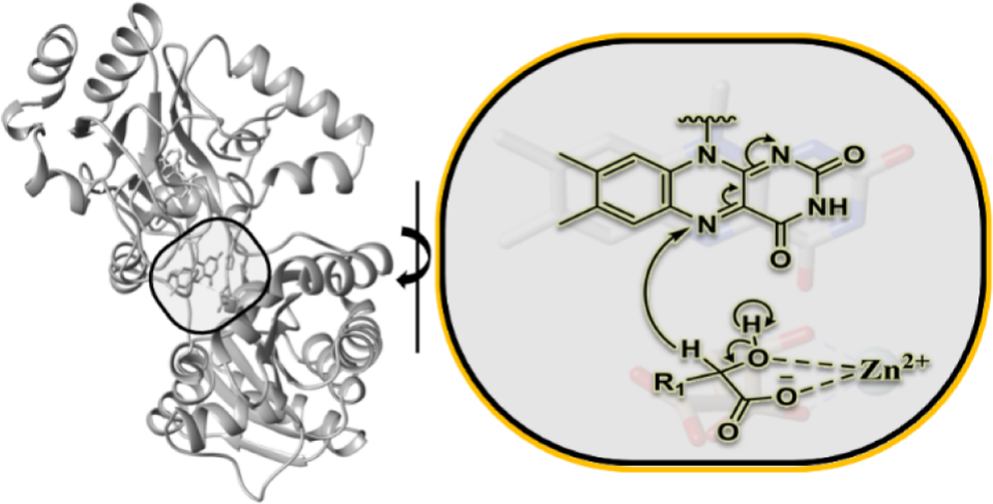

Alcohol oxidation is an indispensable chemical reaction in biological systems. This process, biologically catalyzed by alcohol dehydrogenases (ADHs) and alcohol oxidases (AOXs), follows two distinct chemical routes depending on the cofactor. ADHs have been widely demonstrated to require Zn^2+^- and NAD(P)^+^-based cosubstrates. Except for galactose oxidase, AOXs achieve their conversion of alcohols to aldehydes or ketones using flavin-based cofactors. The FMN-dependent α-hydroxy acid-oxidizing enzymes and the glucose–methanol–choline (GMC) superfamily abstract their substrate’s α–OH proton using a catalytic histidine, leading to substrate oxidation and flavin reduction. However, there is no known alcohol oxidation mechanism for enzymes requiring both a flavin and a metal. The *Pseudomonas aeruginosa*d-2-hydroxyglutarate dehydrogenase (*Pa*D2HGDH) is a recently characterized α-hydroxy acid dehydrogenase that converts d-2-hydroxyglutarate or d-malate to 2-ketoglutarate or oxaloacetate, respectively. *Pa*D2HGDH requires FAD and Zn^2+^ for catalysis. Previous studies on *Pa*D2HGDH have identified a highly conserved active site histidine residue whose position is topologically conserved for catalytic bases in FMN-dependent α-hydroxy acid-oxidizing enzymes and the GMC superfamily of oxidoreductases. In this study, solvent isotope effects (SIEs) coupled with pL-rate profiles and a viscosity control have been used to probe the role of the Zn^2+^ cofactor in the C^2^–OH oxidation of d-malate and flavin reduction of *Pa*D2HGDH. The data revealed an inverse solvent equilibrium isotope effect (SEIE) of 0.51 ± 0.09 consistent with a Zn^2+^-triggered abstraction of the substrate C^2^–OH proton that initiates d-malate oxidation and flavin reduction. The system provides insights into the role of Zn^2+^ in the oxidation mechanism of *Pa*D2HGDH and, by extension, metallo flavoprotein dehydrogenases.

## Introduction

Alcohols are diverse molecules with unique chemical properties and versatile reactivity, making them indispensable in chemistry, biology, pharmacy, and industry.^1^ The versatile oxidation of alcohols to aldehydes, ketones, carboxylic acids, or esters using oxidants like AgNO_3_, K_2_CrO_4_, or KMnO_4_ is a fundamental transformation in organic chemistry, allowing for the synthesis of several complex molecules. The mechanism of alcohol oxidation typically involves the formation of an intermediate species, like a chromate ester or a transition metal complex, followed by an elimination reaction or a rearrangement to form the oxidized product.^1^ The pathway of alcohol oxidation primarily depends on the reaction conditions and the availability of hydrogen atoms on the functional carbon.^2,3^ Primary alcohols, having two hydrogen atoms on the functional carbon, are converted to aldehydes, with subsequent oxidation to carboxylic acids. Secondary alcohols, having only one hydrogen atom on the functional carbon, are oxidized to ketones. Tertiary alcohols, lacking hydrogen atoms on the functional carbon, fragment to yield molecules with new functional groups under harsh conditions.^1^

**Scheme 1:**
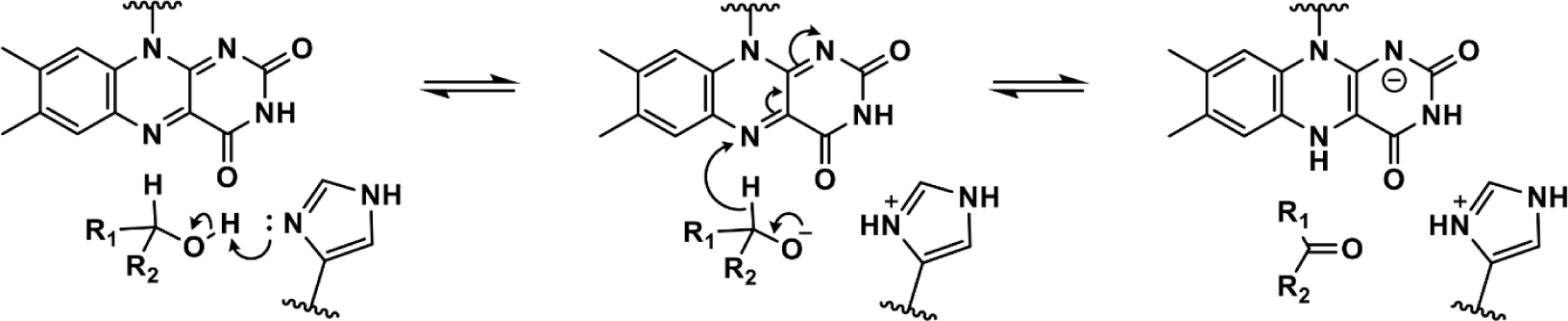
General Reaction Mechanism of Alcohol Oxidation by Alcohol Oxidases and the FMN-Dependent α-Hydroxy Acid Oxidizing Enzymes A conserved active site histidine abstracts a proton from the hydroxyl group, which triggers substrate oxidation and flavin reduction to yield the carbonyl product and reduced flavin cofactor. In alcohol oxidases, R_1_ is either H or an alkyl group, and R_2_ is an alkyl group. In the FMN-dependent α-hydroxy acid oxidizing enzymes, R_1_ is either H or an alkyl group, and R_2_ is a carboxylate group.

**Scheme 2:**
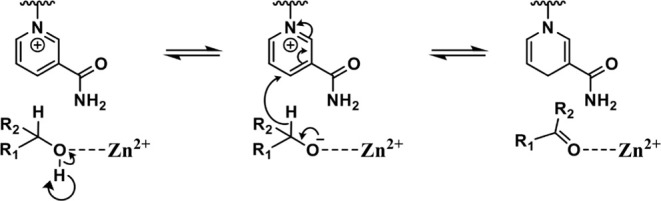
General Reaction Mechanism of Alcohol Oxidation by Alcohol Dehydrogenases (ADHs) An active site metal polarizes the hydroxyl group and is responsible for the OH proton abstraction. The OH proton is lost to an active site residue or the bulk solvent. The alkoxide formation triggers substrate oxidation and the reduction of the nicotine-based cosubstrate NAD(P)^+^, yielding the carbonyl product and NAD(P)H. The circular arrow used for the C^2^–OH group depicts a proton abstraction with an unknown acceptor. Depending on the enzyme system, the proton acceptor can either be the bulk solvent or an active site residue.

Alcohol oxidation is an essential and indispensable chemical reaction central to metabolic pathways like glycolysis and gluconeogenesis, the Krebs cycle, xenobiotic metabolism, and lipid metabolism in biological systems.^4^ Biocatalysis presents an environmentally friendly and renewable alternative to alcohol oxidation.^5^ Enzymes are selective catalysts, allowing precision chemistry without tedious protection group chemistry.^5^ Most studies on alcohol oxidizing enzymes demonstrate the need for metals, flavins, or pyrroloquinoline quinone (PQQ) as cofactors for alcohol oxidation.^4^ For several decades, the catalytic mechanism of various alcohol oxidizing enzymes in biological systems has been investigated, including the flavin-dependent vanillyl alcohol,^6−9^ aryl alcohol,^10−16^ and choline oxidases,^4,17−20^ nonheme iron-dependent oxygenases,^21^ Cu(II)-dependent galactose oxidase,^22^ and the PQQ-dependent ethanol dehydrogenase.^23^ The newer PQQ-containing class of alcohol dehydrogenases employ an aspartate or glutamate for the alcohol OH proton abstraction, leading to PQQ reduction and substrate oxidation.^23,24^ The two major groups of enzymes commonly known to catalyze alcohol oxidation in biological systems are alcohol dehydrogenases (ADHs) and alcohol oxidases (AOXs).^25^ While ADHs have been widely demonstrated to require Zn^2+^ and NAD(P)^+^-based cosubstrates, AOXs achieve their conversion of alcohols to aldehydes or ketones using flavin-based cofactors.^25^ Typically, a conserved active site histidine abstracts a proton from the substrate OH group, triggering flavin reduction and formation of the carbonyl product in AOXs (Scheme 1).^25^ In contrast, ADHs employ an active site metal to abstract the substrate OH proton, leading to NAD(P)^+^ reduction to form NAD(P)H and substrate oxidation to yield the carbonyl product (Scheme 2).^26^

Another group of alcohol-oxidizing enzymes is the FMN-dependent α-hydroxy acid-oxidizing enzymes that catalyze the oxidation of the α-hydroxy acid substrate to a keto acid product.^27^ The catalytic mechanism of the FMN-dependent α-hydroxy acid-oxidizing enzymes involves abstracting their substrate’s C^2^–OH proton using a fully conserved active-site histidine.^28^ The substrate OH proton abstraction triggers substrate oxidation and flavin reduction (Scheme 1). Similarly, the Glucose-Methanol-Choline (GMC) superfamily, sharing a unique fold and a highly conserved active site, oxidizes alcohols using a highly conserved active site histidine, which abstracts a proton from the substrate OH group, initiating substrate oxidation and flavin reduction (Scheme 1).^28^ There has been a clear distinction between the ADH catalytic mechanism of alcohol oxidation, which requires Zn^2+^ and NAD(P)^+^, and the FMN-dependent α-hydroxy acid and GMC-type dehydrogenase mechanism of alcohol oxidation, which requires a histidine and flavin cofactor. To date, a mechanism for alcohol oxidation by an alcohol dehydrogenase with an active site histidine requiring both a flavin-based cofactor and metal has not been established.

**Scheme 3:**
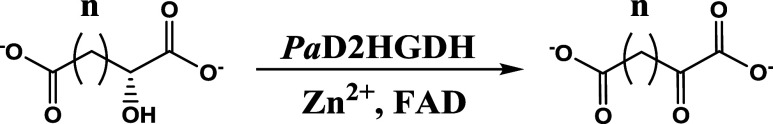
*Pa*D2HGDH Catalytic Scheme *n* = 1 for d-malate and oxaloacetate. *n* = 2 for D-2-hydroxyglutarate and 2-ketoglutarate.

**Figure 1:**
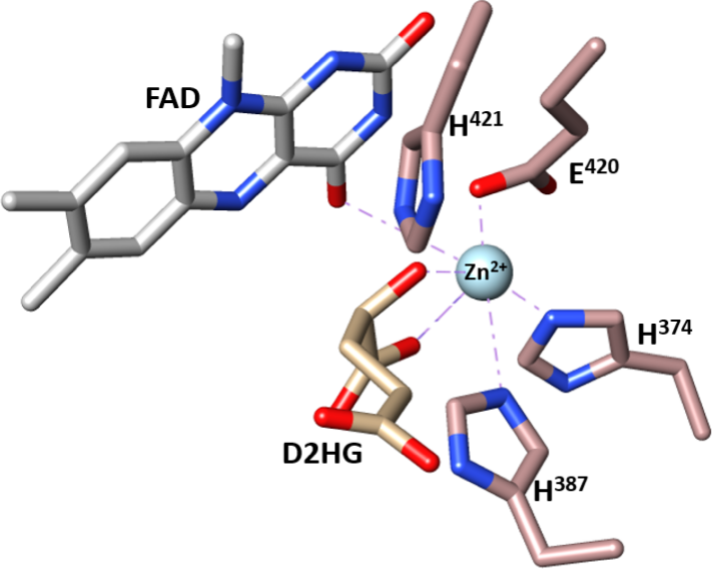
Active site of *Pa*D2HGDH showing the FAD and Zn^2+^ cofactors and the conserved active site residues. The *Pa*D2HGDH homology model was built with SWISS-MODEL using a putative dehydrogenase from *Rhodopseudomonas palustris* (Protein Data Bank code: 3PM9) as a template. The substrate D2HG and Zn^2+^ metal were obtained by the structural overlay of *Pa*D2HGDH and its human homologue (PDB 6LPP). The isoalloxazine ring of the FAD cofactor is shown in gray; the Zn^2+^ cofactor is shown as a cyan sphere; the substrate D2HG is shown in tan; the active site residues are shown in brown; nitrogen atoms are shown in blue; oxygen atoms are shown in red. The protein structures were visualized using UCSF Chimera.^36^

The *Pseudomonas aeruginosa*d-2-hydroxyglutarate dehydrogenase (*Pa*D2HGDH) is a recently characterized enzyme that converts the C^2^–OH in d-2-hydroxyglutarate to a carbonyl group, yielding 2-ketoglutarate as a product.^29−32^ The enzyme is also active with d-malate as an alternative substrate to yield oxaloacetate (Scheme 3).^31^*Pa*D2HGDH plays a vital role in *P. aeruginosa* by maintaining 2-ketoglutarate levels during bacterial l-serine biosynthesis.^33,34^ Knockout of the *Pa*D2HGDH gene has been demonstrated to inhibit the growth of *P. aeruginosa*.^33,34^*Pa*D2HGDH has been shown to use FAD and Zn^2+^ as required cofactors (Figure 1), without which the enzyme is inactive.^29,30^*Pa*D2HGDH is different from other ADHs in that, although its physiological electron acceptor is unknown, it does not use O_2_ as an electron acceptor in the oxidative half-reaction.^29,31^ Previous studies on *Pa*D2HGDH have identified highly conserved amino acid residues common to other α-hydroxy acid oxidizing enzymes.^31^ From a *Pa*D2HGDH homology model built with SWISS-MODEL using a putative dehydrogenase from *Rhodopseudomonas palustris* [Protein Data Bank (PDB) entry 3PM9] as a template, these highly conserved amino acids are located in the enzyme’s active site (Figure 1). A histidine residue is one such conserved active site amino acid whose position is topologically conserved for catalytic bases in the FMN-dependent α-hydroxy acid and GMC-type superfamily of oxidoreductases.^31^ Additionally, the homology model revealed a Zn^2+^ binding site comprising the flavin C^4^O atom and the fully conserved active site amino acids H^374^, H^380^, and E^420^.^30,31^ The active site topology of the *Pa*D2HGDH homology model is similar to the active site of the published crystal structure of human D2HGDH, which has fully conserved H^434^, H^441^, E^475^, and the flavin C^4^O atom as its Zn^2+^ ligands.^31,35^

Previous *Pa*D2HGDH studies have demonstrated a Zn^2+^-mediated lowering of a Zn^2+^-hydrate p*K*_a_ value to ∼7.0, yielding a Zn^2+^-hydroxide species in the ligand-free form of the enzyme.^30^ In the ligand-bound form, the water molecule coordinating the Zn^2+^ cofactor is replaced by the alcohol substrate. Given the Zn^2+^-mediated polarization of water in the substrate-free enzyme, there is a likely polarization of the substrate C^2^–OH upon substrate binding during enzyme catalysis.^29^ Thus, the question remains whether the mechanism of alcohol oxidation in *Pa*D2HGDH follows the classical FMN-dependent α-hydroxy acid/GMC-type catalysis requiring an active site histidine and flavin (Scheme 1), ADH-type catalysis requiring a metal (Scheme 2) or a new catalytic process that is unique for a metallo flavoprotein, comprising a combination of both mechanisms. With both FAD and Zn^2+^ as cofactors and a conserved active site histidine, *Pa*D2HGDH is an excellent candidate for elucidating the catalytic mechanism of a metallo flavoprotein alcohol dehydrogenase.

In the study described herein, solvent isotope effects (SIEs) have been used to probe the role of the Zn^2+^ cofactor in the C^2^–OH oxidation of d-malate and flavin reduction of *Pa*D2HGDH. The results provide insights into the oxidation mechanism of alcohols catalyzed by the Zn^2+^ and FAD-dependent *Pa*D2HGDH and, by extension, metallo flavoprotein dehydrogenases with a GMC-type conserved active site histidine.

## Results

### Rapid-Reaction Kinetics of *Pa*D2HGDH with d-Malate as a Substrate

**Figure 2:**
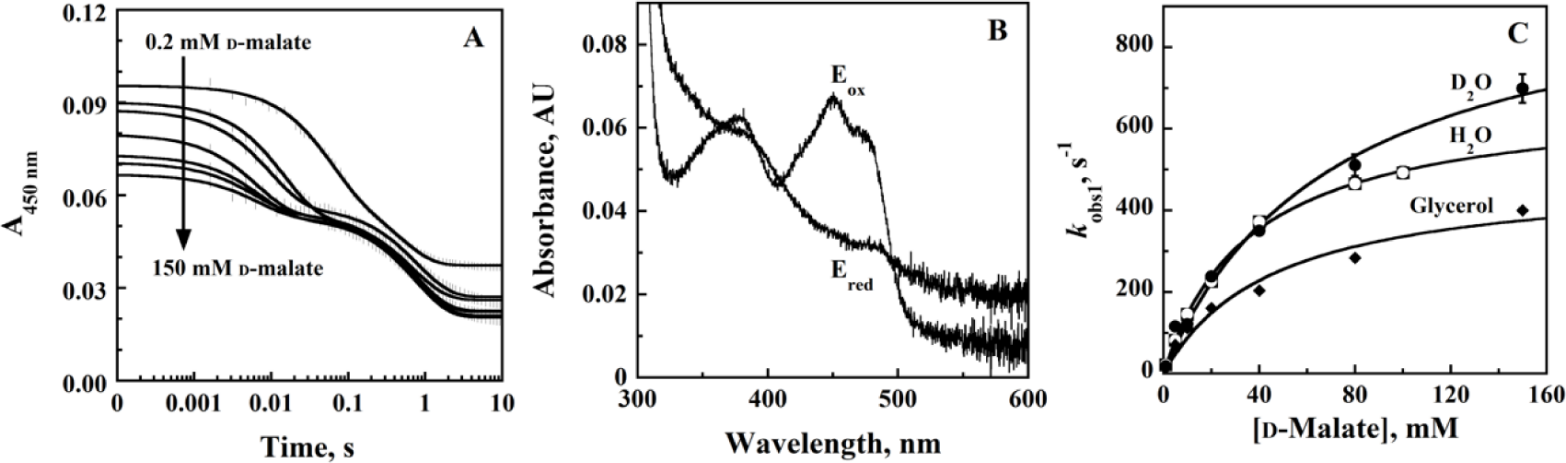
Anaerobic reduction of *Pa*D2HGDH with d-malate as substrate. (A) Stopped-flow traces of *Pa*D2HGDH at 450 nm with varying concentrations of d-malate (0.2–150 mM) fit to eq 4. Note the log time scale. In the interest of clarity, 1 out of every 10 experimental points is shown (vertical lines). Each trace is the average of triplicate runs at each substrate concentration. (B) Absorption spectra of *Pa*D2HGDH showing the fully oxidized flavin before reduction (*E*_ox_) and the fully reduced flavin after reduction with 150 mM of d-malate (*E*_red_). (C) The dependence of *Pa*D2HGDH *k*_obs1_ on d-malate concentration fit to eq 5. The single point shown at each substrate concentration is the *k*_obs_ value obtained from the fit of the average of triplicate runs with eq 4, yielding an error of ≤5%. Data points for the *k*_obs1_ values in H_2_O are shown as ○, those in D_2_O are shown as ●, and those in glycerol are shown as ⧫. Assays were carried out in 0.1 M ACES, 0.052 M Tris, 0.052 M ethanolamine, and 1 mM ZnCl_2_ using an SF-61DX2 Hi-Tech KinetAsyst high-performance stopped-flow spectrophotometer thermostated at 25 °C and equipped with a photomultiplier detector under anaerobic conditions. The instrumental dead time is 2.2 ms. Only the representative data at pL 10.0, having the glycerol control, are shown since all traces, spectra, and plots in protiated and deuterated buffered solutions across all pL values followed similar trends.

To determine the rate of flavin reduction of the metallo flavoprotein *Pa*D2HGDH, the time-resolved anaerobic reduction of *Pa*D2HGDH with d-malate was investigated under pseudo-first-order conditions by monitoring the loss of absorbance of the oxidized flavin at 450 nm at varying d-malate concentrations from 0.2 to 150 mM. The resulting stopped-flow traces showed 2 to 4 distinct reaction phases; however, only the dominant phase with the largest magnitude depended on d-malate concentration. The stopped-flow traces were fit with double, triple, or quadruple exponentials (eqs 2–4) depending on the number of phases in the traces (Figure 2A) to obtain the exponential decay rates for each phase (*k*_obs1_ to *k*_obs4_) at each substrate concentration. The UV–visible absorption spectrum at the end of each reaction showed characteristic properties of a fully reduced *Pa*D2HGDH-bound flavin, as previously reported, showing a charge transfer band between the Zn^2+^ cofactor and the reduced flavin (Figure 2B).^29^

The dominant phase of the time-resolved anaerobic reduction of *Pa*D2HGDH with d-malate, characterized by the bleaching of the oxidized flavin absorption at 450 nm, was assigned to flavin reduction. To obtain the limiting rate constant for flavin reduction (*k*_red_), the exponential decay rates for the dominant phase (*k*_obs1_) assigned to flavin reduction at any given substrate concentration were fit with eq 5, yielding a zero y-intercept hyperbolic dependence of the *k*_obs1_ parameter on d-malate concentration (Figure 2C). The concentration at which half the rate of flavin reduction is recorded (*K*_d_) was also determined from the curve.

### pL Effects on the Rate of Flavin Reduction of *Pa*D2HGDH with d-Malate as a Substrate

To investigate the effect of pH on the *Pa*D2HGDH flavin reduction rate, the anaerobic reductive-half reaction assays were carried out in buffered solutions from pH 6.0 to pH 10.0. The resulting stopped-flow traces showed more reaction phases with increasing pH. The data were fit with the appropriate equations having the correct number of exponential terms corresponding to the number of phases in the stopped-flow traces. At each pH, only the dominant phase depended on d-malate concentration, and there was a zero y-intercept hyperbolic dependence of the *k*_obs1_ parameter on d-malate concentration after fitting the data with eq 5. The resulting *k*_red_ and *K*_d_ kinetic parameters are reported in Table 1.

**Table 1:** Effect of pL on the Rapid-Reaction Kinetic Parameters of *Pa*D2HGDH with d-Malate^a^

pL	(s^–1^)	(s^–1^)	(s^–1^)	(mM)	(mM)	(mM)
10.0	680 ± 20	950 ± 90	490 ± 50	36 ± 3	67 ± 16	46 ± 13
9.5	570 ± 70	800 ± 40		20 ± 6	32 ± 4	
9.0	360 ± 20	900 ± 50		5.0 ± 1.0	7.6 ± 1.2	
8.5	340 ± 30	450 ± 30		3.6 ± 0.7	5.8 ± 0.9	
8.0	200 ± 10	280 ± 10		3.1 ± 0.5	1.8 ± 0.4	
7.5	100 ± 10	120 ± 10		3.4 ± 0.7	3.1 ± 0.7	
7.0	33 ± 2	52 ± 3		2.9 ± 0.6	2.8 ± 0.6	
6.5	7.5 ± 0.4	16 ± 1		3.7 ± 0.7	5.4 ± 0.7	
6.0	1.6 ± 0.1	3.0 ± 0.1		0.7 ± 0.1	1.5 ± 0.2	

a Effect of pL on the rapid-reaction kinetic parameters *k*_red_ and *K*_d_ of *Pa*D2HGDH with d-malate. Each value is shown with the associated standard error from fitting the kinetic data with eq 5. Assays were carried out in 0.1 M ACES, 0.052 M Tris, 0.052 M ethanolamine, and 1 mM ZnCl_2_ at 25 °C. d-Malate was varied from 0.2 mM to 150 mM, with enzyme concentration ranging from 5.5 μM to 7.3 μM to maintain pseudo-first-order conditions.

When the solvent was changed from H_2_O to D_2_O, and the reaction was repeated in buffered solutions from pD 6.0 to pD 10.0 to investigate the solvent isotope effect on the rate of flavin reduction, higher rates of flavin reduction were observed across all pD values (Table 1). There were no observed differences in the overall stopped-flow traces and enzyme spectra in the deuterated buffered solutions compared to the protiated buffered solutions. Additionally, there were no significant differences in the values of the *K*_d_ parameter in protiated and deuterated buffered solutions between pL (pH or pD) 6.5 and 9.0, with greater differences at the pL extremes. Given that the *k*_red_ kinetic parameter (*k*_3_) remains constant above pL 9, the increased *K*_d_ parameter above pL 9 can be explained as either a decrease in the rate constant of free substrate and enzyme association to form the enzyme–substrate (ES) complex (*k*_1_) or an increase in the rate constant of substrate dissociation from the ES complex to yield free substrate and enzyme species (*k*_2_). However, the data do not allow for an unequivocal interpretation of the *K*_d_ pL profile.

**Figure 3:**
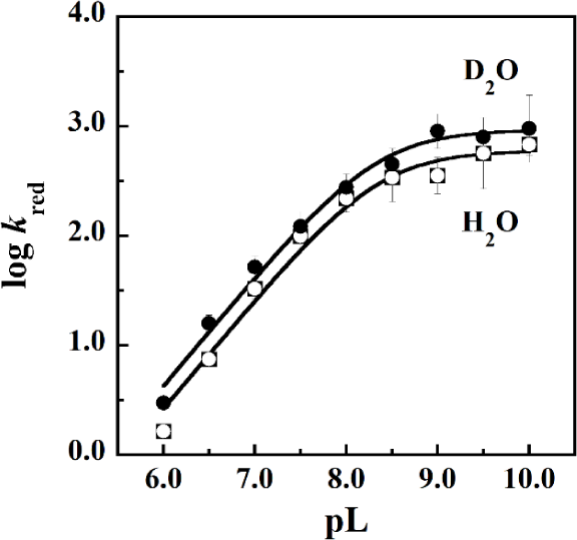
pL dependence of the solvent isotope effects on the rate of *Pa*D2HGDH reduction with d-malate as substrate. Data points for *k*_red_ in H_2_O are shown as ○, and those in D_2_O are shown as ●. Values were determined using the rapid-reaction kinetics approach with varying concentrations of d-malate as a substrate for *Pa*D2HGDH between pL 6.0 and 10.0 under anaerobic conditions. The curves were obtained by fitting the kinetic data to eq 6.

The plot of the log *k*_red_ values as a function of pL fit with eq 6 showed an increase in the *k*_red_ parameter from low to high pL and plateaued above pL 9.0. The observed p*K*_a_ value was 8.3 in both protiated and deuterated buffered solutions, and the pL-independent limiting value (C_H_) at high pL was 600 s^–1^ in H_2_O and 930 s^–1^ in D_2_O (Figure 3 and Table 2).

**Table 2:** Properties of the pL Profile Plots of the Anaerobic Reduction of *Pa*D2HGDH with d-Malate^a^

Property	H_2_O	D_2_O
Limiting value (C_H_), s^–1^	600 ± 100	930 ± 110
p*K*_a_ value	8.3 ± 0.1	8.4 ± 0.1

a Values were extrapolated from the pL profile plots obtained by fitting the kinetic data of the log *k*_red_ parameter as a function of pL using eq 6 upon *Pa*D2HGDH reduction with d-malate as a substrate between pL 6.0 and 10.0 under anaerobic conditions.

### Effect of D_2_O Viscosity on the SIE of *Pa*D2HGDH Flavin Reduction with d-Malate

To investigate the contribution of the higher D_2_O viscosity on the observed deuterium isotope effects on *Pa*D2HGDH flavin reduction, a control viscosity experiment was carried out by adding 9.67% glycerol, having the same relative viscosity as D_2_O, to all enzyme, buffer, and substrate solutions at pH 10.0. The control experiment was run at pH 10.0 since it falls in the pL-independent region of the plot of the log(*k*_red_) parameter against pL to ensure there was no pL effect on the observed isotope and viscosity effects. The data showed an effect of the D_2_O viscosity on the solvent isotope effect measurement.

At pL 10.0, the fastest *k*_red_ value was recorded in D_2_O, yielding a limiting rate constant for flavin reduction of 950 s^–1^. The second fastest *k*_red_ was recorded in H_2_O with a value of 680 s^–1^, followed by the glycerol control having a *k*_red_ value of 490 s^–1^ at pL 10.0 (Table 1 and Figure 2C). The isotope effect on the observed rate of flavin reduction measured in D_2_O  yielded a value of 0.71. However, the glycerol control, having the same relative viscosity as D_2_O, yielded a normal viscosity effect (^Gly^*k*_red_) of 1.38, consistent with an underestimation of the SIE arising from partial masking of the isotope effect by the viscosity effect of the deuterated buffers. Thus, the actual value for the SIE  is given by the ratio of the isotope effect value  to the glycerol effect value (^Gly^*k*_red_), yielding a corrected SEIE  value of 0.51 (Table 3). The computed inverse SIE is comparable to the values reported for other metalloenzymes like thermolysin, carbonic anhydrase, and yeast alcohol dehydrogenase that require metals for substrate–OH proton abstraction.^37,38^

**Table 3:** Solvent Isotope Effect on the Rate of Flavin Reduction in *Pa*D2HGDH with d-Malate^a^

	^Gly^*k*_red_	
0.71 ± 0.08	1.38 ± 0.17	0.51 ± 0.09

a The solvent isotope effect  was calculated by dividing the *k*_red_ kinetic parameter obtained in H_2_O by that obtained in D_2_O. The glycerol effect (^Gly^*k*_red_) was calculated by dividing the *k*_red_ kinetic parameter obtained in H_2_O by that obtained in H_2_O with 9.67% glycerol. The corrected solvent isotope effect  is the given by the ratio of the isotope effect value  to the glycerol effect value (^Gly^*k*_red_). The standard deviation for each value was calculated by multiplying the value determined for the experimental parameter by the square root of the sum of the squares of the percent errors contributed by each experimental parameter.

The three d-malate concentration-independent phases (*k*_obs2_, *k*_obs3_, and *k*_obs4_) in the stopped-flow traces during *Pa*D2HGDH flavin reduction might be due to multiple enzyme–substrate complex species interconverting and not in rapid equilibrium, with only one E_ox_S species being competent to yield the E_red_P complex. The alternative explanation that the other three phases result from damaged enzyme species during the reaction preparation could not be ruled out, although it seemed less likely as typically, in our hands, flavin-dependent enzymes subjected to anaerobic flavin reduction yield at the most up to 10% of the enzyme being damaged in the experiment setup.

Due to the lack of a dependence of the *k*_obs2_, *k*_obs3_, and *k*_obs4_ values on d-malate concentration, the rate constants of the *Pa*D2HGDH isomerization steps *k*_iso1_, *k*_iso2_, and *k*_iso3_ values were computed from the averages of all the *k*_obs2_, *k*_obs3_, and *k*_obs4_ values, respectively, across all d-malate concentrations at each pL value tested (Table S1). There was no observed SIE on the various *k*_iso_ values when the solvent was changed from H_2_O to D_2_O, and there was no significant change in each *k*_iso_ value upon varying the pL from 6.0 to 10.0 (Table S1). Therefore, the averages of the *k*_iso1_, *k*_iso2_, and *k*_iso3_ values across all pLs were computed, yielding rate constants of ∼30 s^–1^ for *k*_iso1_, ∼ 3 s^–1^ for *k*_iso2_, and ∼0.5 s^–1^ for *k*_iso3_, irrespective of the solvent being H_2_O or D_2_O (Table S1). Additionally, no viscosity effect was observed on the rate constants for the isomerization steps.

## Discussion

This study aimed to investigate the role of the *Pa*D2HGDH Zn^2+^ cofactor in the enzyme’s flavin reduction and the C^2^–OH oxidation of d-malate using solvent isotope effects coupled with pL profile studies. When H_2_O is replaced with D_2_O in solvent isotope effect (SIE) studies, three outcomes are expected: (i) an altered equilibrium of ionizable groups involved in enzyme catalysis upon replacing H_2_O with D_2_O. This altered equilibrium is observed as an altered p*K*_a_ value in D_2_O compared to H_2_O, (ii) a viscosity effect arising from the higher viscosity of D_2_O than that of H_2_O, and (iii) a solvent isotope effect arising from the isotopic substitution of the hydrogen in H_2_O with the deuterium in D_2_O.^37,39−42^ The SIE could either be a primary kinetic isotope effect (PKIE) or a solvent equilibrium isotope effect (SEIE). A PKIE originates from the cleavage of the OH bond and arises from the weakened donor-H/D bond in the transition state, yielding a normal effect, i.e., a PKIE > 1; a SEIE originates from the fractionation factors of the species in equilibrium with the H/D and arises from the relative isotopic populations between two ground states in equilibrium, yielding either normal or inverse effects, i.e., SEIE < 1.^37^ To determine the absolute contribution of the deuterium isotope on the kinetic measurement, conclusions must only be drawn in the pL-independent region of the SIE pL profile, which is devoid of pL effects.^37^ Additionally, a viscosity control must be carried out to rule out any viscosity effects from replacing H_2_O with D_2_O.^37,38^ The SIE data from this study demonstrate that Zn^2+^ abstracts the C^2^–OH proton from d-malate and triggers flavin reduction during substrate oxidation catalyzed by *Pa*D2HGDH. Moreover, an internal isomerization precedes flavin reduction during *Pa*D2HGDH catalysis. Details on the Zn^2+^-triggered flavin reduction and d-malate oxidation mechanism in *Pa*D2HGDH are discussed below.

### The Zn^2+^ Cofactor Is Responsible for the C^2^–OH Proton Abstraction during *Pa*D2HGDH Substrate Oxidation

**Scheme 4:**
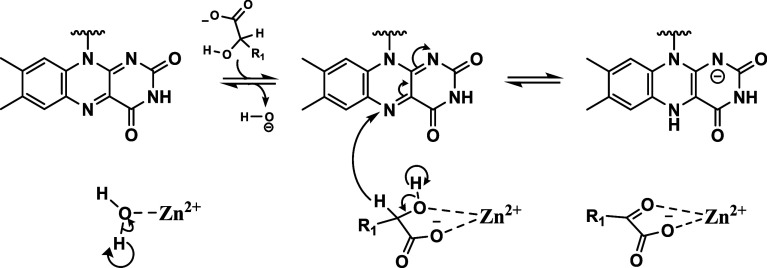
Proposed Mechanism of *Pa*D2HGDH Flavin Reduction and d-Malate Oxidation The binding of d-malate displaces the active site Zn^2+^-hydrate/hydroxide. Zn^2+^ abstraction of the D-malate C^2^–OH proton triggers flavin reduction and D-malate oxidation to yield oxaloacetate. For simplicity, the C^2^–OH proton abstraction and hydride transfer are shown in one step; however, the data do not demonstrate whether these processes are concerted or sequential. The circular arrow used for the water OH and substrate C^2^–OH groups indicates that the proton acceptor is unknown. For *Pa*D2HGDH, the proton acceptor is likely an active site residue.

Evidence to support this conclusion comes from the solvent isotope studies on the anaerobic rapid reaction kinetics of *Pa*D2HGDH with d-malate as a substrate. The results presented in Figure 3 and Tables 2 and 3 and show the presence of an inverse SIE. Due to the inverse nature of the SIE on the rate of flavin reduction, a PKIE can be ruled out. Thus, the observed inverse SIE on the *k*_red_ parameter of *Pa*D2HGDH, having a value of 0.51 after correction for the viscosity effect in D_2_O, is consistent with a SEIE (given by the ratio of the reactant fractionation factor, ϕ, to the product fractionation factor) in which there is a Zn^2+^-mediated abstraction of the C^2^–OH proton, which triggers flavin reduction during *Pa*D2HGDH catalysis of d-malate. Considering that the catalytic step of flavin reduction is partially rate-limiting in *Pa*D2HGDH and the enzyme’s reverse commitment to catalysis is non-negligible,^29^ an inverse SEIE is expected for a Zn^2+^-mediated substrate C^2^–OH deprotonation, which is required for flavin reduction. Conventionally, solvent isotope effects coupled with pL profile studies demonstrate the roles of catalytic metals in enzymes.^37,39−41,43^ An observed inverse SIE would establish the presence of a metal-hydroxide/alkoxide arising from the rapid-equilibrium transfer of a metal-polarized proton in a rate-limiting step during catalysis.^37,40,43,44^ Due to the partial positive charge on oxygen, when water or alcohol (A) is coordinated to Zn^2+^, the fractionation factors, ϕ, of the Zn^2+^-hydrate/alcohol (ϕA), describing the preference of exchangeable groups OH or OD for D or H (eq 1), are inverse and the reactant ϕ is smaller than that of the product.^37^ Thus, the observed inverse SEIE can be explained as a rapid dissociation of the Zn^2+^-hydroxide and equilibration of the Zn^2+^-alcohol substrate that triggers the loss of the C^2^–OH proton upon d-malate binding to yield the Zn^2+^-alkoxide, as shown in Scheme 4.^43,45^

A similar mechanism involving substrate polarization by Zn^2+^ was previously proposed for *Pa*D2HGDH after observing a charge transfer band arising from Zn^2+^ polarization of a Zn^2+^-hydrate species to yield a Zn^2+^-hydroxide species in the ligand-free form of *Pa*D2HGDH.^29^ However, the pL profiles of the *k*_cat_ and *k*_cat_/*K*_m_ kinetic parameters were bell-shaped, and the p*K*_a_ values for the protonated and unprotonated groups were less than 2 pL units apart, preventing the determination of a pL-independent region. Thus, an SIE could not be computed to allow the assignment of Zn^2+^ as the proton abstractor.^29,30^ In contrast, systems that use active site amino acids like histidine for proton abstraction, such as glucose oxidase, pyranose-2-oxidase, choline oxidase, and cholesterol oxidase, yield normal or no SIEs when there is a proton in flight involved in the transition state of the substrate–OH bond cleavage or if the active site preorganization facilitates the formation of an alkoxide intermediate before substrate binding, respectively, since the C^2^–OD group yields a slower rate of flavin reduction in deuterated buffered solutions.^4,40,46−57^
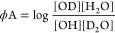
1

### An Unprotonated Group Is Required for *Pa*D2HGDH Oxidation of d-Malate

This conclusion is supported by the reductive half-reaction pL profiles of *Pa*D2HGDH with d-malate as a substrate. The pL profile of the *k*_red_ parameter yielded a plot with a + 1 slope for the increasing limb from low to high pL. The data are consistent with the requirement of an unprotonated group with a p*K*_a_ value of ∼8.3 during substrate oxidation catalyzed by *Pa*D2HGDH. The data favor the assignment of the p*K*_a_ associated with flavin reduction to the ionization of an amino acid side chain of an active site residue. The side chain is protonated at low pL values and cannot accept the Zn^2+^-abstracted C^2^–OH proton, disfavoring catalysis. At high pL values, the competent unprotonated form of the side chain, which can accept the Zn^2+^-abstracted C^2^–OH proton, is favored and dominant, facilitating catalysis. In flavin-dependent enzymes that catalyze alcohol oxidation reactions like choline oxidase, the unprotonated catalytic group has been assigned to a highly conserved active site histidine that acts as a base during substrate oxidation.^19,58^

**Figure 4:**
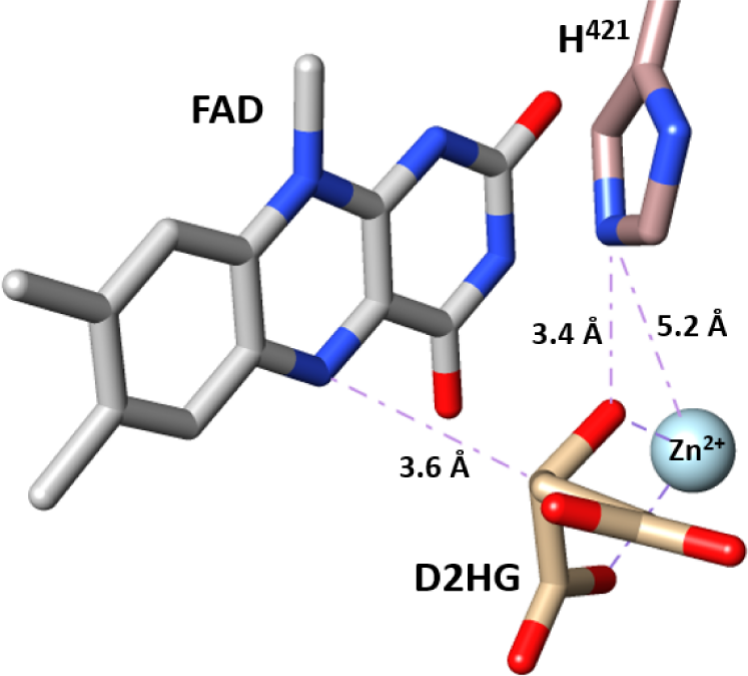
Active site of *Pa*D2HGDH showing the distances and locations of FAD, D2HG, Zn^2+^, H^421^, and E^420^. The *Pa*D2HGDH homology model was built with SWISS-MODEL using a putative dehydrogenase from *Rhodopseudomonas palustris* (Protein Data Bank code: 3PM9) as a template. The substrate D2HG and Zn^2+^ metal were obtained by the structural overlay of *Pa*D2HGDH and its human homologue (PDB 6LPP). The isoalloxazine ring of the FAD cofactor is shown in gray; the Zn^2+^ cofactor is shown as a cyan sphere; the substrate D2HG is shown in tan; the active site residues are shown in brown; nitrogen atoms are shown in blue; oxygen atoms are shown in red. The protein structures were visualized using UCSF Chimera.^36^

Studies on Zn^2+^-dependent metalloenzymes that require a nucleophilic Zn^2+^-hydroxide for catalysis, like carbonic anhydrase, have reported increasing limbs from low to high pLs in the pL profiles of steady-state kinetic parameters, characteristic of an unprotonated group, as observed for *Pa*D2HGDH.^59^ In such Zn^2+^-dependent metalloenzymes, the unprotonated catalytic group has been assigned to the nucleophilic Zn^2+^-hydroxide species, which remains bound in the presence of substrates during enzyme catalysis.^59^ In a previous study on *Pa*D2HGDH, an increasing limb from low to high pL was observed in the *k*_cat_/*K*_m_ pL profile, which reported on the ligand-free form of the enzyme during catalysis and was assigned to a Zn^2+^-hydroxide species in the absence of substrates.^29^ In agreement with the *Pa*D2HGDH published data, a Zn^2+^-hydrate species has been reported in the ligand-free form of the published crystal structure of human D2HGDH. However, in *Pa*D2HGDH, since the Zn^2+^-hydroxide is displaced upon substrate binding^29^ and considering that the *k*_red_ parameter probes the ligand-bound state of the enzyme, the identity of the unprotonated group in the *k*_red_ parameter pL profile being the Zn^2+^-hydroxide is ruled out. A similar explanation involving solvent hydroxide species can be ruled out since that scenario would yield a continuous positively sloped line from low to high pL due to the ever-increasing concentration of hydroxide species with increasing pL. Alternatively, the fully conserved H^421^ residue in the active site of the enzyme is suitably positioned in a geometry and distance that can allow it to act as a proton acceptor^a^ upon the Zn^2+^-triggered proton abstraction from the substrate C^2^–OH group (Figure 4). However, the present data cannot be used to conclude the identity of the unprotonated group required for *Pa*D2HGDH flavin reduction.

The observation of the same p*K*_a_ value for the unprotonated group in the pL profiles of the *Pa*D2HGDH *k*_red_ parameter in the protiated and deuterated buffered solutions can be explained by a compensation of the inverse effect of producing the Zn^2+^-hydroxide ion, having a ∼+0.5 p*K*_a_ shift in D_2_O, by the inverse fractionation factor of the Zn^2+^-bound substrate alkoxide species. Upon binding the Zn^2+^ cofactor, the substrate C^2^–OH group becomes a strong acid, making it easily ionizable. The enzyme uses the Zn^2+^ cofactor to stabilize the substrate alkoxide during the formation of the substrate carbonyl group.^40^ Thus, the substrate C^2^–OH proton likely undergoes a reaction-induced change in its fractionation factor, resulting in an inverse fractionation factor ϕ of ∼0.5, as previously reported for horse liver alcohol dehydrogenase.^60^ Consequently, the positive Zn^2+^-^–^OL p*K*_a_ shift in D_2_O is canceled by the inverse ϕ of the Zn^2+^-alkoxide, leading to a net zero effect. The net-zero effect is observed as similar inflection points in the pH and pD profiles, yielding similar p*K*_a_ values in D_2_O and H_2_O as reported for *Pa*D2HGDH in this study.

### An Internal Isomerization of the Enzyme–Substrate Complex Precedes Flavin Reduction in *Pa*D2HGDH

Evidence supporting this conclusion comes from the kinetic solvent viscosity effects (KSVEs) on the reductive-half reaction of *Pa*D2HGDH at pL 10.0, which affected the *k*_red_ parameter. The data suggest that internal isomerization of the enzyme–substrate complex gates flavin reduction in *Pa*D2HGDH since flavin reduction is a chemical step that does not involve solvent-sensitive protein motions.^61^ Viscosity effects are generally known to have no effects on the chemical steps of catalysis.^61^ However, when protein motions gating flavin reduction occur at rates proportional to the rate of solvent fluctuations, KSVEs are observed on the *k*_red_ parameter.^62^ In *Pa*D2HGDH, flavin reduction is likely gated by enzyme conformational changes arising from substrate binding and the C^2^–OH proton abstraction.^63,64^ Such gated flavin reduction has been previously observed for the flavin-dependent *Neurospora crassa* class II nitronate monooxygenase, and a similar gated electron transfer has been reported for the heme and molybdenum-dependent chicken liver sulfite oxidase.^65,66^

## Conclusions

The mechanism of *Pa*D2HGDH substrate oxidation follows neither the classical FMN-dependent α-hydroxy acid/GMC-type catalysis requiring a flavin cofactor and an active site histidine as the substrate OH proton abstractor (Scheme 1) nor the ADH-type catalysis requiring a nicotine-based cosubstrate and a metal as the substrate OH proton abstractor (Scheme 2). Instead, *Pa*D2HGDH presents a unique blend of both catalytic processes, requiring Zn^2+^ for the substrate OH proton abstraction, a proton acceptor, which is likely the side chain of an active site amino acid residue, and a flavin cofactor (Scheme 4). This study is the first to demonstrate a metal-triggered flavin reduction mechanism in a flavin-dependent enzyme. This mechanism may be unique for the new class of metallo flavoproteins.

This study has elucidated the role of Zn^2+^ in the C^2^–OH oxidation of d-malate during *Pa*D2HGDH catalysis, providing the first catalytic mechanism for a metallo flavoprotein dehydrogenase with a conserved active site histidine. The solvent isotope effects coupled with pL profile studies demonstrate that Zn^2+^-mediates abstraction of the C^2^–OH proton that leads to flavin reduction and substrate oxidation. This mechanism differs from the established mechanism for similar enzymes harboring a conserved active site histidine that abstracts the substrate OH proton for substrate oxidation.^28^ The study demonstrates that in *Pa*D2HGDH, which has both Zn^2+^ and histidine in the active site, Zn^2+^ is responsible for the abstraction of the substrate C^2^–OH proton; however, the ability of H^421^ to gain a proton during catalysis remains a question. Moreover, *Pa*D2HGDH flavin reduction is gated by an internal isomerization of the enzyme–substrate complex. This study is the first to assign a catalytic role to an active site Zn^2+^ in a flavoprotein dehydrogenase. Considering that *Pa*D2HGDH is one of the first-reported Zn^2+^ and FAD-dependent metallo flavoproteins, the study provides an essential foundation for understanding the catalytic mechanisms of other D2HGDH homologues and any FAD-dependent metallo flavoproteins to be discovered in the future. Furthermore, the Zn^2+^-triggered substrate C^2^–OH proton abstraction in the presence of a conserved histidine described in this study provides a basis for more in-depth studies on the catalytic roles of conserved active site histidine residues to prevent their incorrect assignment as catalytic bases in complex enzyme systems.

## Experimental Procedures

*Materials.*d-Malate was purchased from Alfa Aesar (Haverhill, MA). Deuterium chloride (99.5%) and deuterium oxide (99.9%) were from Cambridge Isotope Laboratories, Inc. (Andover, MA). Glycerol, ACES, ethanolamine, Tris, ZnCl_2_, and all other reagents were of the highest commercial purity. Recombinant d-2-hydroxyglutarate dehydrogenase from *Pseudomonas aeruginosa* strain PAO1 (*Pa*D2HGDH) was expressed from plasmid pET20b(+) harboring the PA0317 gene, which was designed in-lab and purchased from GenScript. As described previously,^30^ the protein was purified to homogeneity and stored in 25 mM NaPO_4_, 1 mM ZnCl_2_, pH 7.4.

## Rapid-Reaction Kinetics

To investigate the mechanism of substrate oxidation in the metallo flavoprotein *Pa*D2HGDH, rapid kinetics in protiated and deuterated buffered solutions was carried out on a thermostated SF-61DX2 Hi-Tech KinetAsyst high-performance stopped-flow spectrophotometer equipped with a photomultiplier detector. To maintain a constant ionic strength across all pL values (“L” from lyonium or lyate is used to denote either H or D, depending on the context), the reaction was followed in protiated or deuterated 25 mM Good’s buffer (0.1 M ACES, 0.052 M Tris, and 0.052 M ethanolamine) supplemented with 1 mM ZnCl_2_ at 25 °C and pL 6.0 to 10.0. All pH measurements in D_2_O were determined by adding 0.4 units to the electrode reading to account for the D_2_O effect on the pH electrode. The rate of flavin reduction was measured by monitoring the decrease in absorbance at 450 nm that results from the quenching of the oxidized flavin species upon mixing the enzyme with the substrate. The reductive half-reaction was monitored anaerobically under pseudo-first-order conditions, where the enzyme concentration after mixing with the substrate was ∼8 μM, and d-malate concentrations ranged from 0.1 to 150 mM.

For this experiment, *Pa*D2HGDH was first passed through a desalting PD-10 gel filtration column preequilibrated with either protiated or deuterated buffered solutions at various pL values. The resulting enzyme solution was loaded into a tonometer and subjected to a 25-cycle degassing procedure by alternately flushing with oxygen-free argon and applying a vacuum to render the enzyme solution anaerobic. The anaerobic enzyme was subsequently mounted onto the stopped-flow instrument, which had been subjected to an overnight treatment with an oxygen scrubbing system composed of 5 mM glucose and 1 μM glucose oxidase in 100 mM sodium pyrophosphate at pH 6.0 and room temperature. Substrate solutions were prepared by dissolving d-malate in protiated or deuterated buffered solutions with subsequent pL adjustments. The substrate solutions were then loaded into syringes and flushed for 30 min with argon before being mounted onto the stopped-flow instrument. Additionally, 2 mM glucose and 0.5 μM glucose oxidase were present in all buffers, enzyme solutions, and substrate solutions to remove traces of oxygen.

The effects of the isotopically labeled solvent on the rapid kinetics were determined by alternating solvent isotopomers. The enzyme and d-malate volumes were mixed in the stopped-flow spectrophotometer in single-mixing mode following established procedures with an instrument mixing time of 2.2 ms. The activity was assayed in triplicate for each substrate concentration, and the average value was considered. Typically, measurements differed by ∼5%.

## Data Analysis

The time-resolved flavin reductions at each pL in protiated and deuterated buffered solutions were fit to eqs 2–4 depending on the number of phases observed in the stopped-flow traces, which describes a quadruple exponential process for flavin reduction. Here, *k*_obs1_, *k*_obs2_, *k*_obs3_, and *k*_obs4_ represent the exponential decay rates for each phase during the reduction of the enzyme-bound flavin at any given substrate concentration at 450 nm. *A* represents the absorbance at 450 nm at any given time, *B*_*1*_, *B*_*2*_, *B*_*3*_, and *B*_*4*_ are the amplitudes of the absorption changes, *t* is time, and *C* is the absorbance at an infinite time that accounts for the nonzero absorbance of the fully reduced enzyme-bound flavin.
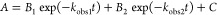
2
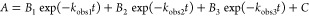
3
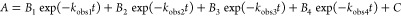
4

The resulting kinetic parameters of the reductive half-reaction were determined after fitting the exponential decay rate of the dominant phase, which was assigned to flavin reduction, at various d-malate concentrations with eq 5. The equation defines a hyperbolic saturation of the enzyme with d-malate, yielding a *y*-intercept value of zero. The data were fit with the KaleidaGraph software (Synergy Software, Reading, PA). Here, *k*_obs1_ represents the exponential decay rate for reducing the enzyme-bound flavin at any substrate concentration (*S*). *k*_red_ is the limiting first-order rate constant for flavin reduction at saturating substrate concentrations. *K*_d_ is the concentration of substrate at which half the limiting rate of flavin reduction is measured and is given by (*k*_2_*+k*_3_)/*k*_1_. The same data was obtained when an equation that defines a hyperbolic saturation with a finite *y*-intercept was used.
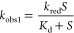
5

The effects of pL on the flavin reduction of *Pa*D2HGDH were investigated by plotting the log values of the *k*_red_ in protiated and deuterated buffered solutions as a function of pL using eq 6. The equation describes a curve that increases with increasing pL with a slope of +1 and a pL-independent limiting value (*C*_L_) at high pL.
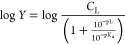
6

## Data Availability

All data are contained within the manuscript except that enclosed in the Supporting Information.

## Supporting Information Available

Isotope and pL effects on enzyme isomerization steps (Table S1) (PDF)

## Abbreviations

*Pa*D2HGDH: *Pseudomonas aeruginosa*d-2-hydroxyglutarate dehydrogenase
D2HG: d-2-hydroxyglutarate
SIE: solvent isotope effect
SKIE: solvent kinetic isotope effect
SEIE: solvent equilibrium isotope effect
KSVE: kinetic solvent viscosity effect.

## Footnotes

a According to the IUPAC Gold Book, a base is a chemical species with an available pair of electrons capable of forming a covalent bond with a proton or with the vacant orbital of another species.^67−69^ The term “catalytic base,” which does not exist in the IUPAC Gold Book, was coined as a convenient way for enzymologists to describe a chemical species that can act (i) as a catalyst by abstracting a proton during enzymatic reactions and (ii) as a base by forming a covalent bond with the abstracted proton. When acting as a catalytic base, a histidine side chain doubles as the catalyst and the proton acceptor. When alcohol proton abstraction is catalyzed by a metal cofactor like Zn^2+^, the proton released by the OH group requires an acceptor, which can be either the bulk solvent or an active site residue like a histidine if the ES complex has geometric constraints shielding solvent access to the OH group.
