# Supplementary material for: Metal-Triggered FAD Reduction in d-2-Hydroxyglutarate Dehydrogenase from Pseudomonas aeruginosa PAO1
Source: ACS Bio Med Chem Au. 2024 Dec 6;5(1):204–14. doi: 10.1021/acsbiomedchemau.4c00108 (PMC11843331; doi:10.1021/acsbiomedchemau.4c00108)
Supplement: Supplementary file 1 — bg4c00108_si_001.pdf [file bg4c00108_si_001.pdf]

Supporting Information for: **Metal-triggered FAD reduction in D-2-hydroxyglutarate dehydrogenase from *Pseudomonas aeruginosa* PAO1**

Joanna Afokai Quaye[1] and Giovanni Gadda[1,2,3,*]

[1]Departments of Chemistry, [2]Biology, and [3]The Center for Diagnostics and Therapeutics, Georgia State University, Atlanta, GA 30302-3965

Corresponding author

*To whom correspondence should be addressed: Giovanni Gadda, Department of Chemistry, Georgia State University, P.O. Box 3965, Atlanta, GA 30302-3965

Phone: (404) 413-5537

FAX: (404) 413-5505

EMAIL: ggadda@gsu.edu

Keywords: *Pseudomonas aeruginosa* D-2-hydroxyglutarate dehydrogenase, solvent isotope effects, viscosity effect, Zn^{2+}, metallo flavoprotein, flavin.

Table S1. Effect of pL on the isomerization steps of *Pa*D2HGDH with D-malate.

pL	k_{iso1} (s^{-1})		k_{iso2} (s^{-1})		k_{iso3} (s^{-1})	
	H_2O	D_2O	H_2O	D_2O	H_2O	D_2O
**10.0**	-	-	3.9 ± 0.7	1.9 ± 0.2	-	-
**9.5**	53 ± 16	14 ± 2	7.9 ± 3.6	2.1 ± 1.3	1.3 ± 0.2	1.8 ± 0.4
**9.0**	8 ± 5	37 ± 8	2.4 ± 0.3	3.3 ± 0.7	0.29 ± 0.03	0.8 ± 0.3
**8.5**	18 ± 2	34 ± 1	2.7 ± 0.5	4.7 ± 0.7	0.24 ± 0.05	0.43 ± 0.07
**8.0**	16 ± 4	23 ± 8	2.1 ± 0.2	2.0 ± 0.2	0.31 ± 0.06	0.7 ± 0.2
**7.5**	-	-	2.7 ± 0.2	3.0 ± 0.4	0.23 ± 0.02	0.34 ± 0.08
**7.0**	-	-	2.1 ± 0.2	2.5 ± 0.3	0.24 ± 0.05	0.33 ± 0.08
**6.5**	-	-	-	1.64 ± 0.03	0.5 ± 0.1	0.5 ± 0.1
**6.0**	-	-	-	0.5 ± 0.1	-	-
**Average**	24 ± 18	27 ± 12	3.4 ± 1.9	2.4 ± 1.8	0.44 ± 0.20	0.70 ± 0.47

Effect of pL on the rapid-reaction kinetic isomerization steps of *Pa*D2HGDH with D-malate. Due to the lack of a dependence of the k_{obs2}, $k_{obs3,}$ and k_{obs4} values on D-malate concentration, the tabulated k_{iso} values were obtained by averaging all the k_{obs} values at the different D-malate concentrations obtained from the fit of the kinetic data with Equations 2-4 depending on the number of observed exponential phases in the stopped-flow traces. k_{iso1}, the average of the k_{obs2} values; k_{iso2}, the average of the k_{obs3} values; and k_{iso3}, the average of the k_{obs4} values. Each k_{iso} value is shown with the associated propagated standard error from the data processing. Assays were carried out in 0.1 M ACES, 0.052 M Tris, 0.052 M ethanolamine, and 1 mM $ZnCl_2$ at 25 ^{o}C. D-Malate was varied from 0.1 mM to 150 mM, with enzyme concentration ranging from 5.5 μM to 7.3 μM to maintain pseudo-first-order conditions.
